# Recent Advances in Thermochemical Water Splitting for Hydrogen Production Using Mixed Ionic-Electronic Conducting Membrane Reactors

**DOI:** 10.3390/membranes15070203

**Published:** 2025-07-04

**Authors:** Jingjun Li, Qing Yang, Jie Liu, Qiangchao Sun, Hongwei Cheng

**Affiliations:** 1 State Key Laboratory of Advanced Refractories, Shanghai University, Shanghai 200444, China; lijingjun@shu.edu.cn (J.L.); 24722465@shu.edu.cn (Q.Y.); 24820363@shu.edu.cn (J.L.); 2 School of Materials Science and Engineering, Shanghai University, Shanghai 200444, China

**Keywords:** hydrogen energy, hydrogen production by water splitting, mixed-conducting oxygen transport membrane, catalyst, membrane reactors

## Abstract

Under the accelerating global energy restructuring and the deepening carbon neutrality strategy, hydrogen energy has emerged with increasing strategic value as a zero-carbon secondary energy carrier. Water electrolysis technology based on renewable energy is regarded as an ideal pathway for large-scale green hydrogen production. However, polymer electrolyte membrane (PEM) conventional water electrolysis faces dual constraints in economic feasibility and scalability due to its high electrical energy consumption and reliance on noble metal catalysts. The mixed ionic-electronic conducting oxygen transport membrane (MIEC–OTM) reactor technology offers an innovative solution to this energy efficiency-cost paradox due to its thermo-electrochemical synergistic energy conversion mechanism and process integration. This not only overcomes the thermodynamic equilibrium limitations in traditional electrolysis but also reduces electrical energy demand by effectively coupling with medium- to high-temperature heat sources such as industrial waste heat and solar thermal energy. Therefore, this review, grounded in the physicochemical mechanisms of oxygen transport membrane reactors, systematically examines the influence of key factors, including membrane material design, catalytic interface optimization, and parameter synergy, on hydrogen production efficiency. Furthermore, it proposes a roadmap and breakthrough directions for industrial applications, focusing on enhancing intrinsic material stability, designing multi-field coupled reactors, and optimizing system energy efficiency.

## 1. Introduction

To address climate change challenges such as global warming, the transition toward low-carbon emission reduction has become a critical global trend. The world’s energy structure is shifting from a fossil fuel-dominated system to a diversified landscape centered on renewable energy. Hydrogen energy, as the most promising clean secondary energy source, has been prioritized as a viable alternative to carbon-based fuels due to its renewability and pollution-free nature [1,2,3,4]. Currently, the international community is striving to resolve the paradox between greenhouse gas emissions and energy security through two approaches: carbon reduction policies and technological innovation. While conventional hydrogen production from fossil fuels (e.g., steam reforming, coal gasification) offers mature techno-economic feasibility, its associated high CO_2_ emissions (12–19 kg CO_2_/kg H_2_) severely hinder sustainable development goals [5,6,7]. Therefore, developing environmentally friendly and sustainable alternatives is imperative. Accordingly, renewable hydrogen production technologies have evolved in three major directions: electrochemical pathways centered on water splitting, thermochemical routes based on biomass conversion, and innovative photocatalytic splitting methods [8,9,10,11,12,13]. Water-splitting hydrogen production technology has garnered great attention due to its feedstock universality and environmental friendliness. Among these technologies, high-temperature thermochemical cycle-based hydrogen production, which utilizes concentrated solar power (CSP) systems to achieve reaction temperatures of 800–2000 °C, can significantly enhance energy conversion efficiency (theoretical > 40%) [14,15,16,17]. However, this approach faces engineering challenges related to reactor material stability and sealing under extreme thermal conditions. In contrast, water electrolysis, when coupled with photovoltaic and wind power systems, has reached commercial maturity, yet its energy efficiency (60–80%) remains constrained by electrode kinetics and system integration limitations. Notably, proton exchange membrane (PEM) electrolysis, despite its advantages in dynamic response and hydrogen purity, still encounters scalability barriers due to the high cost of noble metal catalysts. Moreover, Perfluorosulfonic acid (PFSA) polymer-based proton exchange membranes are the most used low-temperature PEMs in fuel cell applications. However, their poor conductivity under anhydrous conditions, high manufacturing costs, and performance degradation at high temperatures significantly limit their applications. Metal–organic framework (MOF) membranes are crystalline porous materials with a periodic network structure formed by the self-assembly of metal ions or clusters with organic ligands through coordination bonds. During the crystal growth process, it is easy to produce grain boundary defects, pinholes, or cracks, which lead to a decrease in gas permeation selectivity and far lower strength than inorganic ceramic membranes. Table 1 summarizes the current performance limits of various membrane types (e.g., maximum H_2_ production rate, stability hours), including oxygen transport membranes, polymer membranes, and proton exchange membranes.

As early as the 1960s, Funk and Reinstrom researchers proposed the use of thermochemical cycle-based water splitting for hydrogen production [18]. Over the past few decades, this technology has achieved significant advancements due to its clean, abundant, and renewable characteristics. The fundamental principle involves introducing one or more intermediate substances into water to initiate a series of independent yet interconnected chemical reactions, ultimately utilizing thermal energy to decompose water into hydrogen and oxygen. These intermediates remain unconsumed during the reaction process. Compared to direct water thermolysis, this method significantly reduces the required reaction temperature and effectively overcomes the thermodynamic barriers of direct water splitting. Researchers have proposed using metal oxides as intermediates to enable cyclic reactions under moderate temperatures through chemical looping concepts or oxygen transport membranes (OTMs) [19,20,21]. Among these approaches, MIEC membranes involving water-splitting reactions (WSR) have opened a promising pathway by integrating hydrogen production and separation in a single unit while simultaneously conducting oxygen ions and electrons [22,23]. These materials function as selective oxygen exchange media, permitting only oxygen ions and electrons to permeate. Metal oxides serve as oxygen carriers to achieve thermochemical water splitting primarily through redox cycles, theoretically achieving 100% oxygen selectivity. For example, a perovskite-type membrane material (Ba_0.5_Sr_0.5_Co_0.8_Fe_0.2_O_3-δ_) has been reported to exhibit the highest oxygen permeability and selectivity [24,25]. In addition, extensive fundamental research has been conducted by scholars worldwide, covering various aspects such as the development of synthesis methods for ceramic powders, design strategies for membrane chemical compositions and crystal structures, modeling of oxygen permeation, and coupled applications in membrane reactors [26,27].

**Table 1:** Different membrane types are at the current performance limits [28,29,30].

Membrane Types	Working Principle	Maximum H_2_ Production Rate	Maximum Stability Hours
Oxygen transport membrane	Directed migration of oxygen ions	17.4 mL min^−1^ cm^−2^	532 h
Polymeric membrane	Selective separation of ions	600 mmol h^−1^ m^−2^	100 h
Proton exchange membrane	Directed migration of protons	12.6 L h^−1^	3500 h

Research on thermochemical water-splitting hydrogen production technologies is increasingly converging on membrane reactor systems, with their core advantage lying in overcoming both thermodynamic and kinetic limitations through multi-process synergistic effects [31]. The membrane reactor primarily involves three fundamental processes: (1) water dissociation reactions occurring at the catalyst layer and membrane surface on the water side, (2) oxygen diffusion through the bulk membrane phase, and (3) oxidation of reducing gases at the catalyst layer and membrane surface. As an efficient reaction apparatus that integrates membrane separation with catalytic reactions, the membrane reactor demonstrates critical coupling characteristics: insufficient reaction rates on the reduction side reduce the oxygen chemical potential gradient, consequently inhibiting oxygen diffusion, while inadequate water-splitting efficiency directly restricts oxygen supply. Within membrane reactors, catalyst design critically influences both water-splitting efficiency and oxidation kinetics of reducing gases, thereby determining overall reactor performance. Optimizing catalysts requires comprehensive consideration of multiple factors, including activity, stability, selectivity, and membrane compatibility.

**Figure 1:**
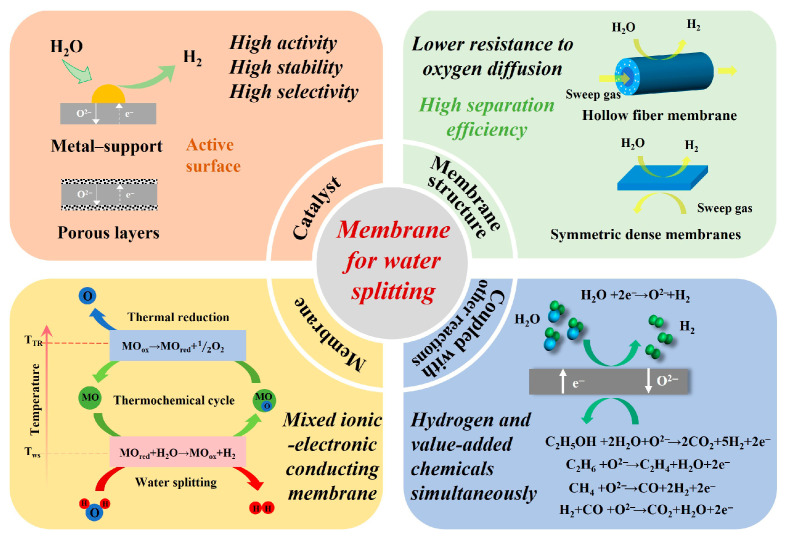
A schematic summary of the main components in this review.

Moreover, introducing reducing gases such as methane, CO, or H_2_ on the opposite side of the membrane can significantly enhance water-splitting conversion rates, facilitating applications of such membrane reactors in hydrogen production via water splitting or hydrogen separation, as shown in Figure 1. This includes the production of high-purity hydrogen and integrated membrane reactors for partial oxidation of methane (POM), oxidative coupling of methane (OCM), ammonia oxidation, CO_2_ reduction, and water-splitting hydrogen production [32,33,34,35,36]. Water splitting reaction, constrained by thermodynamics/kinetics, typically requires elevated temperatures (400–1000 °C for thermally activated transport through dense ceramic membranes [37,38]). While membrane reactors can circumvent thermodynamic limitations through process intensification, catalysts play a pivotal role in determining kinetic performance. Oxygen permeation associated with water splitting comprises surface exchange reactions (including adsorption and dissociation) and bulk diffusion through the membrane. To enhance permeability, both surface exchange reactions and bulk diffusion must be simultaneously promoted [39]. For achieving high hydrogen production rates, MIEC membranes should exhibit high electronic and oxygen ionic conductivity, rapid interfacial reaction kinetics, minimized membrane thickness, and exposure to high oxygen partial pressure (P_O2_) gradients. Researchers have successfully reduced water-splitting temperatures to appropriate levels through thermochemical cycles and catalytic membrane reactors. Notably, despite membrane reactors overcoming traditional thermodynamic equilibrium limitations via process coupling, their practical applications remain constrained by dynamic matching challenges. First, existing membrane materials exhibit exponentially decaying oxygen permeability at intermediate-low temperatures (<700 °C), making them incompatible with conventional industrial heat source temperature ranges. Second, long-term high-temperature operation induces irreversible performance degradation through interfacial element interdiffusion and phase segregation. Last but not least, non-uniform thermal stress distribution and gas bypass issues in scaled-up systems require urgent resolution.

These critical challenges precisely delineate the central research focus on the synergistic optimization of three key elements through materials genome engineering and reactor design innovation. This review systematically consolidates recent advancements in oxygen transport membrane reactors for water splitting and integrated reactions, with focused mechanistic analysis and technological evaluation of three functional domains (catalyst design, membrane structural optimization, and operational parameter modulation) as illustrated in Figure 1. Building upon this foundation, we present a comprehensive perspective on the developmental trajectory, existing challenges, and prospects of high-temperature membrane technology and associated reactors based on water splitting. This work aims to establish both theoretical frameworks and practical pathways for transformative breakthroughs in next-generation high-temperature membrane reactor systems.

## 2. Water Splitting via Membrane Reactors

**Figure 2:**
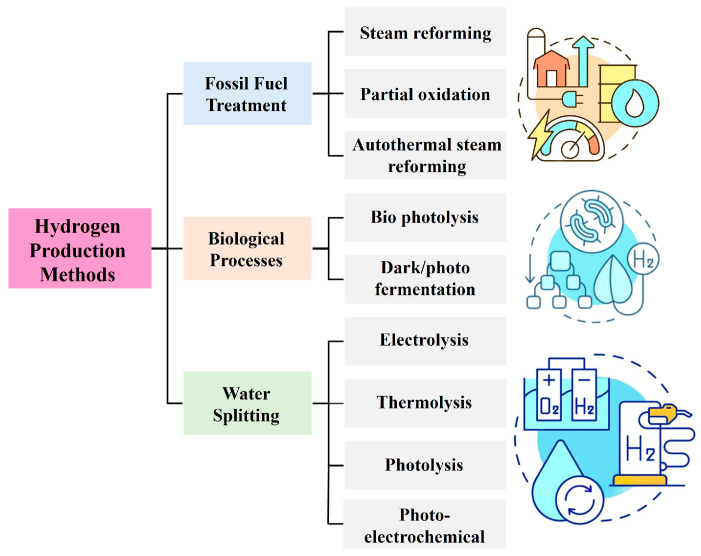
Mainstream hydrogen production methods.

Currently, hydrogen production technologies can be primarily categorized into three methods based on their approaches: fossil fuel-based, biomass-derived, and water-splitting hydrogen production. Figure 2 and Table 2 provide a comprehensive overview of the advantages and disadvantages of these various hydrogen production technologies [40,41,42].

However, fossil fuels such as hydrogen feedstocks face limitations due to their finite reserves and accelerating consumption rates, making it increasingly challenging to meet society’s growing demand. Consequently, future developments in hydrogen production technologies should focus on shifting toward renewable resource utilization. Compared to conventional methods, renewable-based hydrogen production not only offers stable feedstock sources and pollution-free operation but also enables effective integration with renewable energy sources such as solar, tidal, and biomass energy to achieve efficient energy conversion and storage [42,43]. Among these, water represents an ideal hydrogen source due to its abundant availability and renewable characteristics. Water-based hydrogen production technologies can be classified into three main systems: water electrolysis, thermochemical water splitting, and photocatalytic water splitting [10,13]. Thermochemical water splitting (TWS), as a crucial water-based hydrogen production technology, relies on cyclic materials undergoing multi-step thermochemical reactions at elevated temperatures to achieve water splitting for hydrogen production. In chemical looping water splitting processes, oxygen carrier materials facilitate hydrogen production through alternating redox reactions, though this approach results in intermittent and cyclical hydrogen production. To achieve continuous and stable hydrogen production while addressing challenges related to product separation and the alternating operation of oxygen carrier materials, researchers have innovatively proposed OTM reactors that enable continuous water splitting processes [23].

**Table 2:** Major advantages and disadvantages of different hydrogen production technologies [40,41,42].

Method	Advantages	Disadvantages
**Fossil Fuel Treatment** Steam reforming Partial oxidation Thermal steam reforming	Most established industrial process Higher production yields Economically more feasible High-purity hydrogen is produced	Causes massive greenhouse gas emissions Limited feedstock availability High operation temperature requirement Lack of adequate carbon capture and storage Catalyst poisoning
**Thermochemical Processes** Gasification Pyrolysis	Higher production yields CO_2_ neutral Sustainable feedstock supply Short hydraulic retention times Negative CO_2_ emission	High operation temperature requirement. Increased tar formation. Varying H_2_ content
**Biological Processes** Biophotolysis Dark/photo fermentation	Sustainable feedstock availability Negative CO_2_ emission More environmentally friendly Less energy-intensive Provides H_2_ and waste simultaneously	Low production yields and rates Long hydraulic retention times are required Difficulty in maintaining process stability High O_2_ sensitivity Require a large surface area No waste recycling (biophotolysis)
**Water splitting** Electrolysis Thermolysis Photolysis Photoelectrochemical	Sustainable feedstock supply O_2_ is the sole by-product CO_2_ neutral	High investment and operational costs Low conversion efficiency Corrosion problems Sunlight demand (photolysis)

### 2.1. Working Principle of Membrane Reactors

Chemical looping water splitting achieves hydrogen production through a two-step redox cycle reaction. For the oxygen exchange materials (OEMs) constituting oxygen transport membranes, the specific reaction mechanisms are illustrated in Equations (16), where M*_x_*O*_y_* and M represent the metal oxide and metallic state of the OEM, respectively, while *x*, *y*, and δ denote stoichiometric coefficients [44]. During the reduction step, the OEM undergoes either thermal splitting at elevated temperatures or reduction by reducing gases to form reduced-state OEM-red. In the subsequent oxidation step, steam oxidizes OEM-red back to oxidized-state OEM-ox while simultaneously generating pure H_2_. Throughout this cyclic process, only water is consumed, while the OEM maintains its oxygen transfer capability through redox reactions involving lattice oxygen exchange, thereby realizing thermochemical water splitting for hydrogen production [45].

Reduction Process:

(1) $$M_{x}O_{y}\to M_{x}O_{y-\delta }+\frac{1}{2}\delta O_{2},$$

(2) $$M_{x}O_{y}+\delta CH_{4}\to M_{x}O_{y-\delta }+\delta ({CO}_{2}+{2H}_{2}),$$

(3) $$M_{x}O_{y}+\delta CO\to M_{x}O_{y-\delta }+\delta {CO}_{2},$$

(4) $$M_{x}O_{y}+\delta H_{2}\to M_{x}O_{y-\delta }+\delta H_{2}O.$$

Oxidation Process:

(5) $$M_{x}O_{y-\delta }+\delta H_{2}O\to M_{x}O_{y}+\delta H_{2}.$$

Net Reaction:

(6) $$H_{2}O\to \frac{1}{2}O_{2}+H_{2}.$$

However, in conventional chemical looping water splitting processes, the intermittent redox cycling of OEMs leads to discontinuous hydrogen production. To achieve continuous hydrogen generation and address this limitation, researchers have developed an innovative OTM reactor system composed of oxygen exchange materials. This system enables simultaneous redox reactions to occur on opposite sides of the membrane, thereby facilitating the continuous production of pure hydrogen.

**Figure 3:**
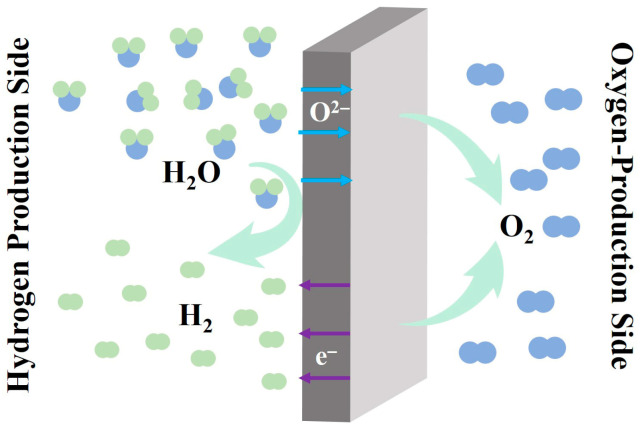
Schematic diagram of the OTM reactor principle.

The OTM reactor utilizes oxygen transport membranes to achieve continuous water splitting. By selecting appropriate oxygen exchange materials for membrane fabrication, the reaction can proceed efficiently and continuously to produce high-purity hydrogen. These OTM materials possess unique MIEC properties at elevated temperatures. As illustrated in Figure 3, the OTM operates through a distinctive mechanism: water vapor is introduced on one side (feed side) of the membrane, while an inert gas (N_2_, He) or reducing gas (CO, CH_4_) is swept across the opposite side (sweep side) [45]. Upon water vapor exposure at the feed side, water molecules dissociate into hydrogen and oxygen species (Equation (7)). When an oxygen chemical potential gradient exists across the membrane, oxygen ions (O^2−^ migrate through oxygen vacancies or interstitial sites in the lattice from the high oxygen partial pressure side to the low oxygen partial pressure side, while electrons move in the opposite direction to maintain charge neutrality [46]. At the sweep side, the transported oxygen ions either recombine to form molecular oxygen (Equation (8)) or react with reducing gases to form new compounds (Equations (9) and (10)) [47].

(7) $$H_{2}O+2e^{-}\to O^{2-}+H_{2},$$

(8) $$2O^{2-}\to O_{2}+4e^{-},$$

(9) $$O^{2-}+\mathrm{CO}\to {\mathrm{CO}}_{2}+2e^{-},$$

(10) $$O^{2-}+CH_{4}\to {CO}_{2}+H_{2}+2e^{-}.$$

The oxygen transport process in membrane materials is governed by two rate-limiting steps: surface oxygen exchange on both sides and bulk diffusion. When the oxygen permeation process is predominantly controlled by bulk diffusion, the oxygen flux through the membrane can be calculated using the Wagner equation [48]:

(11) $$J_{O_{2}}=-\frac{RT}{16F^{2}L}\int_{lnP_{O_{2}}^{\prime }}^{lnP_{O_{2}}^{\prime \prime }}\frac{\sigma_{el}\sigma_{ion}}{\sigma_{el}{+\sigma }_{ion}}dlnP_{O_{2}},$$

where $\sigma_{\mathrm{ion}}$ and $\sigma_{\mathrm{el}}$ are the ionic and electronic conductivities of the membrane (S∙cm^−1^), respectively; the ratio of $\frac{\sigma_{\mathrm{el}}\sigma_{\mathrm{ion}}}{\sigma_{\mathrm{el}}{+\sigma }_{\mathrm{ion}}}$ represents the electronic migration number during oxygen permeation; L denotes the membrane thickness (m); T is the thermodynamic temperature (K); F is the Faraday constant; $P_{O_{2}}^{\prime }\;$ high and $P_{O_{2}}^{\prime \prime }$ low are the oxygen partial pressures on the high and low oxygen partial pressure sides of the oxygen transport membrane (Pa). Since $\sigma_{\mathrm{ion}}$ is much smaller than $\sigma_{\mathrm{el}}$ in most mixed-conducting oxygen transport membrane materials, Equation (5) can be simplified to

(12) $$J_{O_{2}}=-\frac{RT}{16F^{2}L}\int_{lnP_{O_{2}}^{\prime }}^{lnP_{O_{2}}^{\prime \prime }}\sigma_{ion}dlnP_{O_{2}}.$$

Therefore, when bulk diffusion serves as the rate-limiting step, the oxygen permeability of membrane materials is primarily determined by temperature, membrane thickness, oxygen partial pressure gradient across the membrane, as well as ionic and electronic conductivities.

When the oxygen permeation process is predominantly controlled by surface exchange kinetics, the oxygen flux can be calculated using the following equation [49]:

(13) $$J_{O_{2}}=\frac{k_{s}C_{O}}{4RT}∆\mu_{O_{2}}^{Surf}.$$

In this context, $∆\mu_{O_{2}}^{\mathrm{Surf}}$ represents the oxygen chemical potential gradient across the membrane surfaces, $C_{O}\;$ denotes the surface molar concentration of oxygen, and $k_{s}$ is the exchange coefficient of the membrane material. The surface oxygen exchange reaction rate is primarily governed by the intrinsic properties of the oxygen transport membrane.

When the oxygen permeation process is jointly controlled by both bulk diffusion and surface exchange mechanisms, the influence of bulk diffusion on oxygen flux progressively diminishes as membrane thickness decreases. At a critical thickness where the surface exchange resistance becomes comparable to bulk diffusion resistance, the membrane reaches its critical thickness L_c_, defined as

(14) $$L_{c}=D^{*}/k_{s},$$

where $k_{s}$ is the oxygen exchange coefficient of the membrane surface, and $D^{*}$ is the tracer oxygen ion diffusion coefficient. When the thickness of the transport membrane is L > $L_{c}$, the velocity-controlled step is a bulk diffusion reaction, and when L < $L_{c}$, the permeation process is mainly controlled by the surface oxygen exchange reaction.

### 2.2. Composition Optimization of Membrane Materials

OTMs are fabricated from MIEC materials [50], which exhibit both ionic and electronic conductivity. After decades of research, various oxygen transport membranes with promising application potential have been systematically screened. Key evaluation criteria for OTM materials include oxygen permeability, chemical stability, mechanical strength, corrosion resistance, and cost-effectiveness. Currently, fluorite-type and perovskite-type materials have been extensively studied and practically applied, with numerous publications providing detailed characterizations of these materials [51,52,53,54,55,56]. Based on their phase composition, OTMs can be classified into single-phase and dual-phase membranes. Single-phase membranes consist of a homogeneous material phase, typically demonstrating high oxygen flux. However, their poor stability under reducing atmospheres significantly limits their practical applications. To address this limitation, researchers have developed dual-phase membranes, which, as the name suggests, incorporate two distinct material phases. The primary advantage of dual-phase membranes lies in their tunable transport properties, which can be engineered to meet specific application requirements. Furthermore, these membranes generally exhibit superior thermochemical stability and enhanced corrosion resistance, making them suitable for harsh operating conditions. The following sections will provide a detailed discussion of the applications of these two membrane types in water-splitting processes.

#### 2.2.1. Single-Phase Membrane Materials

**Figure 4:**
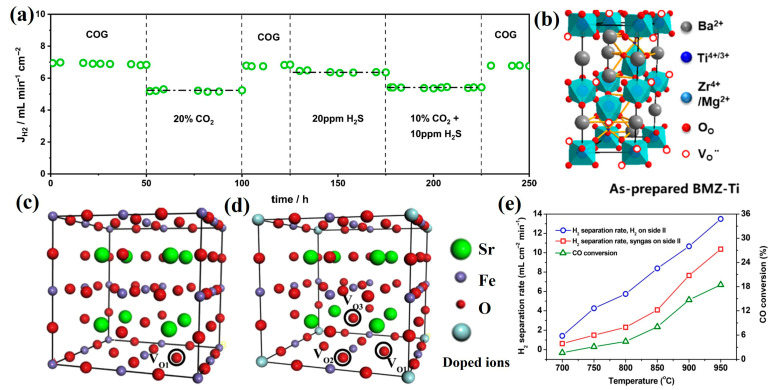
(**a**) Long-term test of asymmetric PSFA CMR under different atmospheres at 900 °C [60]; (**b**) the crystal structure of BaMg_0.1_Zr_0.05_Ti_0.85_O_3-δ_ [61]; the supercells structure diagrams, (**c**) Sr_8_Fe_8_O_24_; (**d**) Sr_8_Fe_7_M_1_O_24_ (M = Al, Zr, Nb, W) [62]; (**e**) the relationship between temperature and H_2_ separation rate and CO conversion [63].

Single-phase membrane materials refer to membranes with a homogeneous phase structure, where both oxygen ions and electrons conduct within the same phase. These materials feature a simple composition and exhibit high oxygen permeability. Researchers have extensively investigated the performance and mechanisms of various single-phase membrane materials in water splitting, with a summary provided in Table 3. Single-phase oxygen transport membranes are primarily categorized into fluorite-type (e.g., CaF_2_), perovskite-type (ABO_3_), and K_2_NiF_4_-type structures [57]. Evdou et al. [58] developed a disk-shaped membrane reactor using La_0.3_Sr_0.7_FeO_3-δ_ (LSF) perovskite, achieving a hydrogen production rate of 0.0145 mL min^−1^ cm^−2^ under reducing conditions enhanced by CO as a reducing agent. Park et al. [59] synthesized La_0.7_Sr_0.3_Cu_0.2_Fe_0.8_O_3-δ_ (LSCF) dense ceramics via conventional solid-state reaction, observing that the hydrogen generation rate increased with decreasing membrane thickness and increasing water vapor partial pressure, P_H2O_. At 900 °C and 49 vol% H_2_O, a 0.33 mm LSCF membrane achieved a peak hydrogen production rate of 1.93 mL min^−1^ cm^−2^. Furthermore, when a 50-μm-thick LSCF layer was coated onto a porous carbon-based support (20 wt% carbon) with Pt porous layers on both sides, the hydrogen production rate reached 11.41 mL min^−1^ cm^−2^ at 900 °C. Ghanem et al. [60] fabricated an asymmetric membrane via sol–gel synthesis, using Pr_0.6_Sr_0.4_Fe_0.9_Al_0.1_O_3-δ_ (PSFA) as the dense layer material. The membrane employed CP-PSFA (10 wt% Ni) as the support/catalytic layer for oxidizing coke oven gas (COG) and Ni/Ce_0.9_Pr_0.1_O_2_ (Ni/CP) as the catalyst for water splitting. At 940 °C, the hydrogen production rate reached 9.8 mL min^−1^ cm^−2^, seven times higher than that of a conventional dense membrane. Notably, this system also facilitated CO-free hydrogen production through water splitting and partial oxidation of methane in COG to syngas. Additionally, as shown in Figure 4a, the membrane demonstrated stable performance for over 250 h under varying atmospheres.

Perovskite materials typically adopt a cubic ABO_3_ structure, where A and B represent cation sites. The A-site is preferentially occupied by alkaline earth or rare earth ions with larger ionic radii, while the B-site is generally filled by transition metal ions with smaller radii. Research on B-site doping modifications has revealed that the incorporation of high-valent dopant ions can effectively modulate the material’s phase structure, reduce oxygen vacancy concentration, and enhance the average metal-oxygen bond energy (ABE), thereby improving material stability. He and his co-authors [61] synthesized Ti-doped BaMg_0.1_Zr_0.05_Ti_0.85_O_3-δ_ (BMZ-Ti) membranes via a citrate-EDTA sol–gel method. As illustrated in Figure 4b, the crystal structure exhibits mixed ionic-electronic conductivity under low oxygen partial pressures, attributed to Mg acceptor doping and the moderate reducibility of Ti^4+^. This membrane demonstrated stable hydrogen production via water splitting at a rate of 0.8 mL min^−1^ cm^−2^, maintaining excellent durability with no observable degradation after 100 h of continuous operation. Liu et al. [62] conducted a systematic investigation of B-site doped perovskite oxygen transport membranes for hydrogen production via water splitting, focusing on microstructure, chemical stability, and hydrogen generation performance. Using sol–gel synthesis, they prepared Pr_0.6_Sr_0.4_Fe_0.9_M_0.1_O_3-δ_ (PSFM, M = Fe, Al, Zr, W) membranes. For oxygen vacancy analysis, the Pr_0.6_Sr_0.4_FeO_3_ unit cell was simplified to SrFeO_3_, constructing a 2×2×2 supercell model (Figure 4c).

In the undoped Sr_8_Fe_8_O_24_ model, only one type of oxygen vacancy (VO_1_) was identified. Upon doping with Al, Zr, or W, the supercell models transformed into Sr_8_Al_1_Fe_7_O_24_, Sr_8_Zr_1_Fe_7_O_24_, and Sr_8_W_1_Fe_7_O_24_, respectively, with three distinct oxygen vacancy configurations (VO_1_, VO_2_, VO_3_) emerging due to different B-site ion-oxygen vacancy distances (Figure 4d). The Pr_0.6_Sr_0.4_Fe_0.9_Al_0.1_O_3-δ_ (PSFA) membrane achieved the highest hydrogen production rate of 1.07 mL min^−1^ cm^−2^ at 900 °C, attributed to its elevated oxygen vacancy content and reduced oxygen vacancy formation energy while maintaining stable performance at 1.0 mL min^−1^ cm^−2^ for 50 h. Li and co-authors [63] fabricated 0.5 mm Ba_0.98_Ce_0.05_Fe_0.95_O_3-δ_ membranes with 1 wt% Ru/Sm_0.15_Ce_0.85_O_1.925_ catalysts applied via brush-coating and impregnation on both surfaces (Figure 4e). The hydrogen production rate increased from 1.4 mL min^−1^ cm^−2^ at 700 °C to 13.5 mL min^−1^ cm^−2^ at 950 °C, demonstrating that controlled doping can effectively tune critical physicochemical properties of perovskite oxygen exchange materials, including phase structure, oxygen vacancy concentration, grain boundary characteristics, and average metal-oxygen bond energy (ABE), thereby enhancing the performance of oxygen transport membranes.

**Table 3:** Summary of the performances of single-phase membranes in the WSR.

Membrane Material	Membrane Structure (thick/mm)	Tem (°C)	Atmosphere on Both Sides	Catalysts at the Water Side	Hydrogen Production Rate (mL min^−1^ cm^−2^)	Stability Performance (h)	Ref.
BMZT	Disk (0.7)	960	He/H_2_O; CH_4_/CO_2_/He/N_2_	—	0.8	100	[61]
LSF	Disk (3)	860	H_2_O; He	—	0.0145	—	[58]
LSF	Disk (3)	860	H_2_O; CO	—	0.0048	—	
LSCF	Disk (0.33)	900	N_2_/H_2_O; H_2_/He	Pt	1.93	—	[59]
LSCF	Thin film (0.05)	900	N_2_/H_2_O; H_2_/He	Pt	11.41	—	
LCF	Thin film (0.5)	990	Ar/H_2_O; He/CH_4_	—	0.2	—	[64]
LCF	Thin film (0.9)	990	Ar/H_2_O; He/CH_4_	—	0.5	—	
BCF	Disk (0.5)	950	N_2_/H_2_O; H_2_/He	Ru-SDC	13.5	100	[63]
BSCF	Disk (0.07)	950	He/H_2_O; CH_4_/He/Ne	—	3.3	—	[65]
BFZ	Disk (1.6)	900	N_2_/H_2_O; CO_2_/He	—	0.3	—	[66]
BFZ	Tubular (1.05)	900	N_2_/H_2_O; CO/He	—	1.99	—	[67]
BZCF	Tubular (0.17)	900	H_2_O; CH_4_	—	2.2	—	[31]
PSFA	Disk (1.0)	900	N_2_/H_2_O; CO/N_2_	—	1.07	50	[62]
PSFA	Disk (1.0)	940	N_2_/H_2_O; CH_4_/H_2_	Ni/CPO	9.8	250	[60]
LNO/GDC10	Double-layer disk	900	H_2_O/N_2_; CO_2_/CO/He	—	0.12	—	[68]
SCFZ	Tubular	900	H_2_O; EtOH	—	3.4	60	[69]
SFC	Thin film (0.02)	900	N_2_/H_2_O; H_2_/He	—	17.4	—	[70]
SFC	Disk (1.04)	900	N_2_/H_2_O; H_2_/He	—	4.0	—	

#### 2.2.2. Dual-Phase Membrane Materials

**Figure 5:**
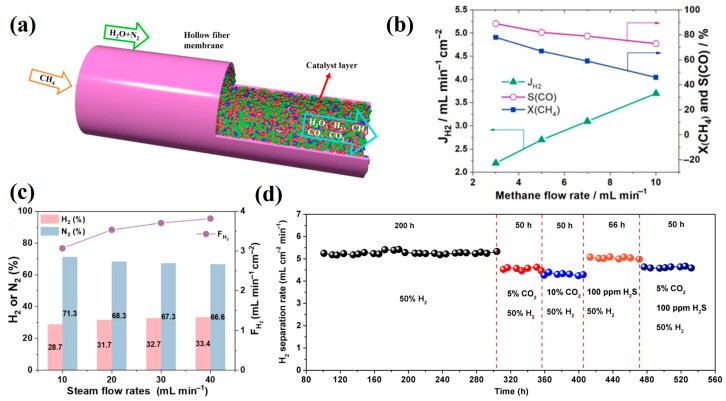
(**a**) Schematic diagram of the LCF-Ag hollow fiber membrane reactor for water splitting coupling with partial oxidation of methane [73]; (**b**) X_CH4_ and J_H2_ as a function of feeding methane flow rate for the CGO membrane reactor WS side: F_H2O_ =  30 mL  min^−1^and F_He_ =  10  mL  min^−1^, POM side: F_Ne_ =  1  mL  min^−1^, F_CH4_ +  F_He_ =  19  mL  min^−1^, T  =  930  °C [74]; (**c**) H_2_O splitting test results using SDC-LCAF membrane reactor with 10 wt% Ni/SDC catalyst on the shell side at 950 °C, using constant H_2_ concentration of 80% in the sweep gas and varying steam flow rates: shell side gas composition and H_2_ production rate [76]; (**d**) long-term operation of the SDC-SFM membrane reactor under various atmospheres at 900 °C [77].

In membrane reactors for water splitting, redox reactions occur on both sides of the membrane, necessitating high material stability. While single-phase membranes offer facile fabrication and high oxygen permeability, their poor stability under reducing atmospheres represents a critical limitation, with some materials suffering rapid structural degradation that impedes long-term operation. The conceptual breakthrough came when Mazanec et al. [71] introduced dual-phase MIEC membranes, spurring extensive research on these materials. Dual-phase membranes, characterized by tunable composition and enhanced stability (detailed in Table 4), have emerged as promising alternatives. The incorporation of metallic phases (e.g., Co or Ni) effectively addresses the intrinsically low electronic conductivity of ceramic dual-phase membranes while maintaining good water-splitting performance. Balachandran et al. [72] provided definitive evidence using 0.13 mm Ni-GDC membranes that hydrogen originates from water splitting rather than permeation/leakage, achieving hydrogen production rates of 6.0 mL·min^−1^ cm^−2^ at 900 °C through porous metal-ceramic layers that enhance the surface area. Zhang et al. [73] developed La_0.8_Ca_0.2_Fe_0.94_O_3-δ_-Ag (LCF-Ag) hollow fiber membranes via sol–gel synthesis combined with phase inversion/sintering. As illustrated in Figure 5a, the reactor employed a Ni/LaNiO_3_/γ-Al_2_O_3_ catalytic system on the permeate side, yielding hydrogen production rates of 3.6 mL·min^−1^ cm^−2^ with 2.5 mL·min^−1^ H_2_ flow in the lumen under H_2_/N_2_ shell-side atmosphere. Liang et al. [74] fabricated cobalt-doped Ce_0.8_Gd_0.2_O_2-δ_ membranes via tape casting and lamination, with surface-impregnated La_0.4_Sr_0.6_CoO_3-δ_ catalysts. The system achieved 90% initial methane conversion, with hydrogen production reaching 3.7 mL·min^−1^ cm^−2^ at 10 mL·min^−1^ CH_4_ flow (Figure 5b). Notably, while Ni-based catalysts are commonly used in combined water splitting/partial methane oxidation systems employing MIEC membranes, they suffer from rapid deactivation via coking when processing methane feeds.

Compared to perovskite structures, fluorite-structured MIEC materials exhibit superior phase stability and minimal chemical expansion, which are crucial for long-term reactor operation. Single-phase oxygen transport membranes based on ion-conducting (IC) fluorite-type CeO_2_ demonstrate excellent oxygen ion mobility and redox properties, though their limited electronic conductivity remains a major constraint. This challenge has been addressed by developing MIEC-IC composite materials that combine both functionalities. The incorporation of Ni catalysts into MIEC-IC composite frameworks significantly increases the number of triple-phase boundary sites for water splitting and partial oxidation of POM reactions compared to unmodified frameworks.

Fang et al. [75] utilized this approach to fabricate a Ce_0.85_Sm_0.15_O_1.925_-Sm_0.6_Sr_0.4_Al_0.3_Fe_0.7_O_3-δ_ dual-phase membrane, achieving an exceptional hydrogen production rate of 11.7 mL·min^−1^ cm^−2^. Remarkably, the membrane maintained stable operation for 100 h under CO-containing reducing atmospheres, demonstrating outstanding chemical stability. Zhang et al. [76] developed a hollow fiber membrane system using Sm_0.2_Ce_0.8_O_2−δ_-La_0.8_Ca_0.2_Al_0.3_Fe_0.7_O_3-δ_ (SDC-LCAF) for coupled water splitting and methane partial oxidation. The reactor featured a 10 wt% Ni/SDC catalyst on the shell side and Ni/LaNiO_3_/γ-Al_2_O_3_ on the lumen side for POM. The system showed no performance degradation after 100 h of continuous operation at 950 °C. Further investigations examined the effects of gas composition and flow rates on reactor performance. As shown in Figure 5c, experiments with a constant 80 vol% H_2_ concentration in the sweep gas and varying steam flow rates (10–40 mL·min^−1^) on the shell side revealed that hydrogen production rates initially increased with the steam flow before stabilizing. The maximum recorded hydrogen production rate reached 3.81 mL·min^−1^ cm^−2^. Cai et al. [77] synthesized a novel hydrogen separation membrane material, Ce_0.85_Sm_0.15_O_1.925_-Sr_2_Fe_1.5_Mo_0.5_O_6-δ_, which exhibits high conductivity under low oxygen partial pressures, facilitating both electron transfer and ion diffusion. A 0.5 mm membrane coated with Ni/SDC catalyst achieved a high hydrogen separation rate of 6.6 mL·min^−1^ cm^−2^ at 900 °C. As illustrated in Figure 5d, the membrane reactor demonstrated exceptional stability, operating continuously for 532 h in atmospheres containing CO_2_ and H_2_S impurities. Future research should focus on designing membrane reactors that simultaneously achieve high water-splitting rates and long-term operational stability.

**Table 4:** Summary of the performances of multi-phase membranes in the WSR.

Membrane Material	Membrane Structure (thick/mm)	Tem. (°C)	Atmospheres on Both Sides	Catalysts at Water Side	Hydrogen Production Rate (mL cm^−2^ min^−1^)	Stability Performance (h)	Ref.
ZrO_2_-TiO_2_-Y_2_O_3_	Tubular (2)	1600	H_2_O; H_2_/CO_2_	—	0.5	—	[78]
Ni-GDC	Disk (0.13)	900	H_2_O; H_2_/He	Ni-GDC	6	—	[72]
Ni-GDC	Disk (0.09)	900	N_2_/H_2_O; H_2_/He	—	3.7	—	[79]
Co-GDC	Disk (0.03)	930	H_2_O/He; CH_4_/He	LSC	1.8	100	[74]
Ag-LCF	Hollow fiber	950	H_2_O/N_2_; H_2_/He	—	3.6	—	[73]
Ag-LCF	Hollow fiber	950	H_2_O/N_2_; CH_4_	—	0.69	—	
SDC-SSAF	Symmetrical disk (0.03)	900	H_2_O; CH_4_	Ni	11.7	100	[75]
SDC-SSAF	Asymmetric disk (0.07)	950	H_2_O/He; H_2_/N_2_	Ru-based catalyst	12.2	—	[80]
SDC-SSAF	Symmetric disk (0.07)	950	H_2_O/He; H_2_/N_2_	Ru-SDC	11.9	—	
SDC-SSAF	Symmetric disk (0.4)	950	H_2_O/He; H_2_/N_2_	Ru-SDC	5.9	—	
SDC-SSCF	Disk (0.36)	900	H_2_O/He; H_2_/N_2_	Ni-SDC	7.5	120	[81]
SDC-SSCF	Disk (0.36)	900	H_2_O/He; H_2_/N_2_	Co-SDC	5.0		
SDC-SSCF	Disk (0.36)	900	H_2_O/He; H_2_/N_2_	Fe-SDC	6.8		
SDC-SSCF	Disk (0.38)	900	H_2_O/He; H_2_/N_2_	Ni-SDC	9	—	[82]
SDC-SSCF	Disk (0.38)	900	H_2_O/He; H_2_/N_2_	Ru-SDC	16	—	
SDC-SFM	Disk (0.5)	900	H_2_O/He; H_2_/N_2_	Ni-SDC	6.6	532	[77]
SDC-SFM	Disk (0.44)	800	H_2_O/Ar; CH_4_/Ar	PNO	4.5	250	[83]
SDC- SFM	Disk	970	H_2_O/He/Ne; CH_4_/ He/Ne	—	1.5	100	[84]
GDC-GSTA	Disk (1.1)	—	H_2_O; H_2_	Ni-GDC	3.1	—	[85]
GDC-GSTA*_δ_*	Disk (1.2)	900	Ar/H_2_/H_2_O; Ar/ H_2_/H2O	—	0.67	—	[86]
CPO-PSMT	Disk	940	H_2_O/He; H_2_/N_2_	—	0.52	180	[87]
CPO-PSFA	Disk (1)	925	H_2_O/N_2_; CO/He	—	1	20	[88]
CPO-PSFA	Disk layer	925	H_2_O/N_2_; CO/He	—	1.2	20	
ZYO-LSCF	Symmetric disk	900	H_2_O/N_2_; CH_4_/He	—	0.40	300	[89]
ZYO-LSCF	Symmetric disk		H_2_O/N_2_; CO/He, N_2_		0.54	300	
YSZ-LSCF-CuO	Disk (0.5)	900	H_2_O/He; CO/H_2_	Ni-SDC	0.76	290	[90]
SDC-LCAF	Hollow fiber	950	H_2_O/CO_2_; CH_4_	Ni-SDC	3.81	100	[76]

### 2.3. Structural Design of Membrane Materials

**Figure 6:**
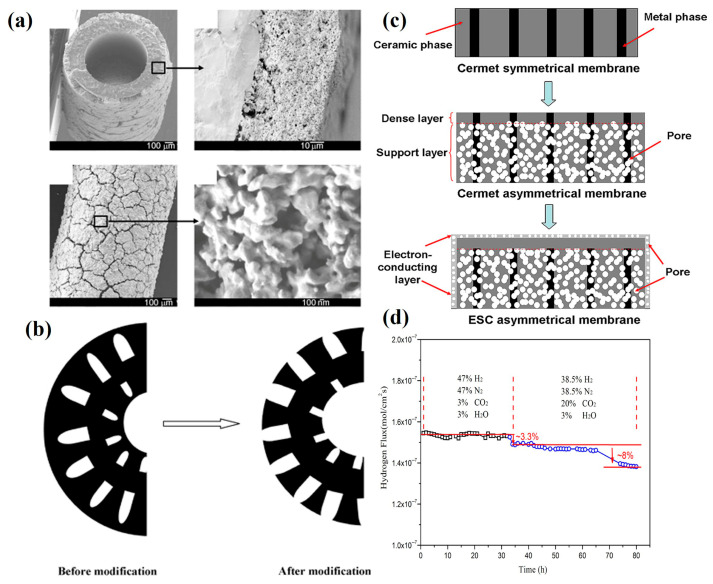
(**a**) SEM micrographs of the BCFZ hollow-fiber membrane with a BCFZ-Pd layer treated at 1050  °C for 1 h [95]; (**b**) schematic of membrane architectures to be improved by the acid etching process [96]; (**c**) three configurations of hydrogen separation membrane-based proton conductor: symmetric membrane; asymmetric membrane; ESC asymmetrical membrane; (**d**) the stability of hydrogen permeation through ESC membrane [98].

Currently, oxygen transport membrane reactors mainly adopt three structural configurations: disk-shaped, tubular, and hollow fiber geometries. While disk and tubular membranes have been extensively utilized in early-stage research due to their relatively simple fabrication processes [91,92,93], these conventional designs present inherent limitations. Disk-shaped membranes in particular exhibit significant thickness-related drawbacks, resulting in comparatively higher oxygen diffusion resistances. This characteristic has been observed in various membrane systems, including CP-PSFA [94] and other membranes. The substantial thickness of these conventional membranes directly impacts their oxygen permeation fluxes and consequently limits reactor performance. Notably, recent advances in membrane structure design have revealed that structural optimization can significantly enhance hydrogen separation efficiency in membrane reactors. Hollow fiber membranes offer the highest membrane surface area per unit packing volume with thin separation layers that enable high oxygen permeation fluxes. As shown in Figure 6a, a BCFZ-Pd layer approximately 40 μm thick is tightly adhered to the outer surface of the BCFZ hollow fiber membrane. The surface-modified BCFZ membrane exhibits a 3.5-fold increase in oxygen permeation flux compared to the unmodified BCFZ membrane [95].

The incorporation of catalyst-impregnated porous layers significantly enhances oxygen surface exchange rates, and this configuration has been widely adopted in most oxygen transport membrane reactors. Li et al. [80] proposed a dense SDC-SSAF membrane with a thickness of 70 μm that demonstrated a hydrogen production rate of 12.2 mL min^−1^ cm^−2^ when modified with a catalyst-impregnated porous layer. Acid etching represents a relatively simple method for creating finger-like pores on both inner and outer membrane surfaces [96], as illustrated in Figure 6b. Cai et al. [97] fabricated an asymmetric SDC-SSCF membrane featuring straight-through channels in the porous layer using tape casting combined with laser grooving technology, achieving approximately 30% improvement in water-splitting performance. Our research group investigated the working mechanism of porous layers by spin-coating a CP porous layer onto PSFA membranes. Under testing conditions at 925 °C, this modification resulted in a 43% increase in oxygen flux to 3.7 mL min^−1^ cm^−2^ [88], demonstrating enhanced oxygen permeability and hydrogen production capability. The Ni–Ba(Zr_0.1_Ce_0.7_Y_0.2_)O_3−δ_ (Ni-BZCY) composite membrane developed by Zhu et al. [98] has been extensively studied for membrane permeability applications. The structural evolution from symmetric to asymmetric configurations, as depicted in Figure 6c,d, represents an effective approach for reducing membrane thickness. While externally short-circuited asymmetric membranes exhibit superior hydrogen permeation performance compared to conventional asymmetric membranes, they demonstrate inadequate hydrogen permeation durability under CO_2_-containing atmospheres.

Shao et al. [99] developed an innovative microchannel ceramic membrane for oxygen separation, featuring numerous parallel microchannels penetrating the ceramic matrix. In this architecture, one terminus of each microchannel remains open while the opposite end terminates at an ultrathin-dense layer. Compared with conventional dense membranes, this novel configuration achieved a remarkable enhancement in oxygen permeation flux, exceeding fivefold. However, the oxygen transport mechanism through modified hollow fiber membranes remains governed by the coupled effects of surface reaction kinetics and bulk diffusion as the rate-limiting steps, particularly under elevated operating temperatures. To further advance membrane performance, future research should prioritize minimization of the dense layer thickness in the hollow fiber core to reduce transport resistance and systematic optimization of the porous layer architecture to enhance gas–solid interactions.

### 2.4. Factors Affecting the Performance of Membrane Materials

According to the Wagner equation, the oxygen permeation flux through membrane materials is primarily determined by four key factors: operating temperature, membrane thickness, oxygen partial pressure gradient across the membrane, and ionic/electronic conductivities [48]. Consequently, the oxygen transport kinetics may be rate-limited by either surface oxygen exchange reactions at the gas–solid interfaces or bulk diffusion processes within the membrane material. In previous studies, two principal strategies have been commonly employed to enhance oxygen permeation performance through material modifications: reduction in membrane thickness to minimize diffusion path lengths and enhancement of ionic conductivity to improve charge carrier transport. These approaches focus on optimizing the intrinsic properties of the membrane materials themselves.

**Figure 7:**
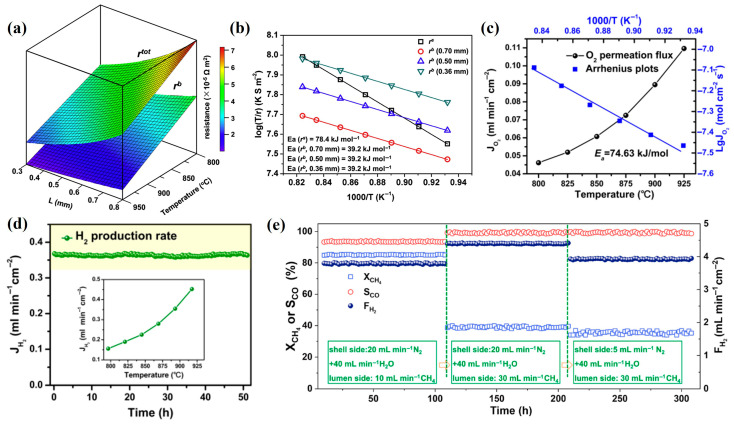
(**a**) Dependence of the total resistance (rtot) and bulk resistance (rb) on the temperature and membrane thickness (L) of the SDC-SSCF membrane; (**b**) Arrhenius plots of the different resistances and the calculated activation energies [81]; (**c**) temperature dependence of oxygen permeation fluxes and corresponding Arrhenius plot; (**d**) the long-term hydrogen production performance of the PSFN membrane at 900 °C (the inset is temperature dependence of hydrogen production rates) [100]; (**e**) long-term stability of LCFCu0.05 HFM coupling WS and POM at different experimental conditions [101].

When studying the water-splitting performance of SDC-SSCF membrane materials with different thicknesses, Cai et al. [81] established a simple model for a membrane reactor coupling water-splitting reaction and hydrogen oxidation reaction, investigated the effects of bulk diffusion resistance and surface reaction resistance, and quantitatively calculated the surface reaction resistances $\left(r^{s}\right)$ of water-splitting reaction and hydrogen oxidation reaction, as well as the bulk diffusion resistance $\left(r^{b}\right)$ of the membrane at different temperatures, as shown in Figure 7a. Since the three membrane reactors have the same catalyst and there is limited data available for modeling studies, $r^{s}$ is used to represent the sum of the water-splitting reaction resistance and the hydrogen oxidation reaction resistance. The total resistance ($r^{\mathrm{tot}}$) can be obtained by the sum of $r^{b}$ and $r^{s}$, i.e., ($r^{\mathrm{tot}}$ = $r^{s}$ + $r^{b}$). Meanwhile, the oxygen partial pressure gradient between the H_2_O(g) side and the H_2_ side is very large (10^5^–10^6^), so it is reasonable to assume that the $r^{s}$ of the three membranes with different thicknesses are similar, and $r^{b}$ is proportional to the membrane thickness L. As shown in Figure 7a, both $r^{b}$ and ${\;r}^{\mathrm{tot}}$ decrease with the increase in temperature. Therefore, $r^{s}$ also follows the same law. At a certain temperature, $r^{b}$ increases linearly with the thickness, and the ratio of $r^{b}$/$r^{\mathrm{tot}}$ (%) reflects the degree to which the hydrogen separation process is controlled by bulk diffusion. For the 0.70 mm membrane, the $r^{b}$/$r^{\mathrm{tot}}$ (%) ratio is greater than 50% when increasing the temperature and membrane thickness, indicating that $r^{b}$ is greater than $r^{s}$ within the studied temperature range. For the 0.36 mm membrane, $r^{s}$ is slightly higher than $r^{b}$. Considering that $r^{b}$ is comparable to $r^{s}$, the hydrogen separation process is jointly controlled by bulk diffusion and surface reaction within the studied thickness range. As shown in Figure 7b, the calculated activation energies of bulk diffusion resistance and surface reaction resistance are 39.2 kJ·mol^−1^ and 78.4 kJ·mol^−1^, respectively. When the membrane thickness decreases, the proportion of surface reaction resistance increases. Temperature is another important factor affecting the oxygen permeation process of membrane materials, and the water splitting rate increases with the increase in temperature. For Ni-GDC membrane materials, when the membrane thickness decreases from 1.70 mm to 0.10 mm, the water splitting rate increases from 1.2 mL·min^−1^ cm^−2^ to 4.3 mL·min^−1^ cm^−2^. When the thickness decreases from 0.28 mm to 0.13 mm, the water splitting rate increases slowly; although the thickness is halved, the water splitting rate only increases by 18% [72]. For the 1 mm PSFN membrane, when the temperature increases from 800 °C to 920 °C, the oxygen permeation amount increases from 0.043 mL min^−1^ cm^−2^ to 0.11 mL·min^−1^ cm^−2^, and the corresponding H_2_ production rate increases from 0.15 mL·min^−1^ cm^−2^ to 0.45 mL·min^−1^ cm^−2^, as shown in Figure 7c,d [100].

The oxygen permeation process in mixed-conducting membranes involves a sequence of surface reactions including molecular oxygen adsorption, oxygen dissociation into atomic species, lattice incorporation of oxygen ions, and product desorption. Each of these elementary steps significantly influences the overall surface exchange kinetics, which is fundamentally determined by the intrinsic properties of the membrane material. To enhance surface oxygen exchange rates, researchers commonly employ surface modification strategies such as increasing membrane surface area through micro-/nanostructuring or incorporating catalytic layers to facilitate specific reaction steps. Zhang et al. [101] conducted a comprehensive 300 h stability test on LCFC hollow fiber membranes with dual catalytic modification, featuring Ni/SDC catalyst on the shell side and Ni/LaNiO_3_/γ-Al_2_O_3_ catalyst on the bore side (Figure 7e). Under baseline operating conditions with a feed stream of 20 mL min^−1^ N_2_ and 40 mL min^−1^ H_2_O, along with 10 mL min^−1^ CH_4_ sweep gas, the membrane achieved a hydrogen production rate of 3.8 mL min^−1^ cm^−2^. When the CH_4_ flow rate was increased to 30 mL min^−1^ while maintaining other parameters, the hydrogen production rate improved by 18.4% to 4.5 mL min^−1^ cm^−2^, demonstrating that elevated CH_4_ concentration enhances performance by reducing permeate-side oxygen partial pressure and increasing the oxygen chemical potential gradient. Further optimization by reducing the N_2_ diluent flow to 5 mL min^−1^ maintained a high hydrogen production rate of 3.9 mL min^−1^ cm^−2^, confirming the positive correlation between the water splitting rate and H_2_O partial pressure. Comparative studies of LCF-Ag membranes revealed the significant impact of catalytic modification, with hydrogen production rates increasing from 0.69 mL min^−1^ cm^−2^ in non-catalyzed systems to 7.9 mL min^−1^ cm^−2^ when using Ni/LaNiO_3_/γ-Al_2_O_3_ catalyst [73]. These results clearly demonstrate that the oxygen removal rate at the permeate side serves as the rate-determining step for overall water splitting efficiency. The research underscores the importance of optimizing catalyst design to accelerate oxygen reduction kinetics and facilitate surface exchange processes while maintaining an appropriate balance between maximizing feed-side water partial pressure and preserving a steep oxygen partial pressure gradient to ensure optimal performance.

## 3. Development of Water Splitting Catalysts

Water-splitting reactions require overcoming substantial thermodynamic energy barriers, where catalysts facilitate the process by providing alternative reaction pathways that reduce the required energy input, thereby enabling efficient water decomposition at lower temperatures. In systems with multiple potential reaction pathways, catalysts can selectively promote the target reaction while suppressing side reactions, consequently enhancing both product purity and yield. Water-splitting catalysts can be classified based on their material composition into noble metal catalysts (such as Pt, Ir, Pd, etc.), transition metal catalysts (such as Ni-based, Co-based, Fe-based, etc.), perovskite-based catalysts, and high-entropy alloys, among others [102].

**Figure 8:**
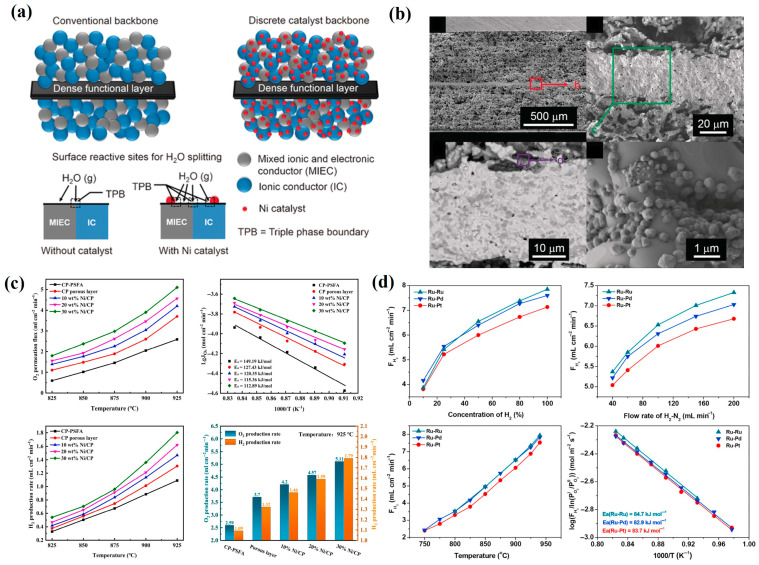
(**a**) Schematic diagrams of activity enhancement of the Ni-infiltrated backbone for H_2_O splitting; (**b**) cross-sectional microstructure of the Ni-infiltrated SDC–SSAF sandwich-like symmetrical dual-phase OTM [75]; (**c**) temperature dependence and Arrhenius plots of the oxygen permeability at reductive atmosphere, the temperature dependence of the hydrogen separation performance, and O_2_ and H_2_ production capacity at 925 °C of CP-PSFA membranes loading of different contents catalyst in the CP porous layer [88]; (**d**) hydrogen separation performance of SSCF-SDC with different M/SDC catalysts coated on the side under different conditions [104].

Early studies by Jiang et al. on BCFZ hollow fiber membrane reactors [31] and Park et al. on LSCF membrane reactors [103] achieved limited water splitting efficiency due to the absence of catalysts on the water dissociation side. To address this limitation, Wei et al. [75] developed an innovative symmetric membrane reactor with a sandwich-like structure, as illustrated in Figure 8a, capable of simultaneously producing hydrogen and syngas at high rates (>10 mL min^−1^ cm^−2^) on opposite sides of an SDC-SSAF membrane. As shown in the SEM image (Figure 8b), this sandwich-structured symmetric membrane comprises a thin, dense layer (~30 μm thick) sandwiched between two thick, porous layers (~500 μm each). The thin, dense layer significantly reduces bulk diffusion resistance for oxygen ion transport, while the porous layers, impregnated with Ni catalyst, substantially increase the number and activity of reactive sites. Performance testing results (Figure 8c) demonstrated that at 925 °C, the CP-PSFA membrane loaded with 30% nickel-based catalyst achieved oxygen permeation and hydrogen production rates of 5.11 and 1.79 mL min^−1^ cm^−2^, respectively, representing 97% and 64% improvements compared to the unmodified membrane [88]. These enhancements can be attributed to the expanded triple-phase boundary area and increased number of surface reaction sites following nickel catalyst loading, which collectively lead to significantly improved membrane separation performance [96].

The majority of membrane reactor studies have focused on nickel-based catalysts, with limited exploration of alternative metal catalysts. Cai et al. [81] systematically evaluated a series of M/SDC (M = Fe, Co, Ni) catalysts applied to both sides of SDC-SSCF membranes. Under identical testing conditions, catalytic performance followed the trend Co-SDC < Fe-SDC < Ni-SDC. The Ni-SDC catalyst demonstrated superior activity, achieving a hydrogen production rate of 7.7 mL min^−1^ cm^−2^ on a 0.36 mm thick SDC-SSCF membrane, representing a 1.5-fold enhancement compared to Co-SDC. Further investigations examined three noble metal catalysts (Ru, Pt, Pd) for both water dissociation and hydrogen oxidation reactions (Figure 8d) [104]. The catalytic activity sequence was established as Ru > Pt > Pd for water splitting and Ru > Pd > Pt for hydrogen oxidation. Comparative studies between Ru/SDC and Ni/SDC catalysts revealed significantly higher activity for Ru/SDC in both reactions. Notably, Ru catalyst implementation doubled the water splitting performance relative to nickel-based systems, achieving an exceptional hydrogen production rate of 21.6 mL min^−1^ cm^−2^ at 900 °C [82]. Jiang et al. [95] demonstrated similar enhancement effects by depositing a BCFZ-Pd porous layer on BCFZ hollow fiber membranes, increasing hydrogen production from 0.7 to 2.1 mL min^−1^ cm^−2^ at 950 °C. While noble metal catalysts (e.g., Pt, Ru) exhibit outstanding catalytic performance, their high costs present significant barriers to large-scale implementation. Consequently, the development of cost-effective, high-efficiency catalysts remains a critical challenge for the commercialization of membrane-based water-splitting technologies.

Perovskite-based catalysts have emerged as promising catalysts for water splitting due to their structural and compositional flexibility, adjustable electronic structure, environmental friendliness, and chemical durability. Wang et al. [105] developed a highly efficient perovskite (La_0.6_Ca_0.4_Mn_0.6_Al_0.4_O_3_) which yielded a remarkable H_2_ production rate of 429 μmol/g during two-step thermochemical water splitting, going between 1000 and 1400 °C. High-entropy oxides (HEO) and high-entropy alloys (HEA) exhibit excellent catalytic performance and corrosion resistance, comparable to that of precious metal catalysts, especially in the fields of electrocatalysis and photothermal catalysis. Zhong et al. [106] prepared the high-entropy oxide catalysts (Ni_0.2_Co_0.2_Ca_0.2_Cu_0.2_Mg_0.2_)Fe_2_O_4_. The fuel was overfed in the fuel reactor to ensure the phase transition from (NiCoCaCuMg)Fe_2_O_4_ to NiCoCuMgFex. During the water splitting in the steam reactor, the excellent H_2_ production performance and anti-carbon deposition performance were displayed. The H_2_ yield of 12.66 mmol/g OC (oxygen carrier) with a purity of 99.1% was exhibited at 700 °C.

## 4. Coupling Reaction Mechanisms Across Membranes

In water-splitting membrane reactors, the sweep side can be coupled with various oxygen-consuming reactions to significantly reduce the oxygen partial pressure, thereby enhancing oxygen permeation rates and driving the water-splitting equilibrium toward hydrogen production. This integrated reactor design enables simultaneous hydrogen generation and the production of value-added chemicals. Reported coupling reactions include hydrogen oxidation, methane partial oxidation, ethane dehydrogenation, and carbon monoxide oxidation [62,64,107].

**Figure 9:**
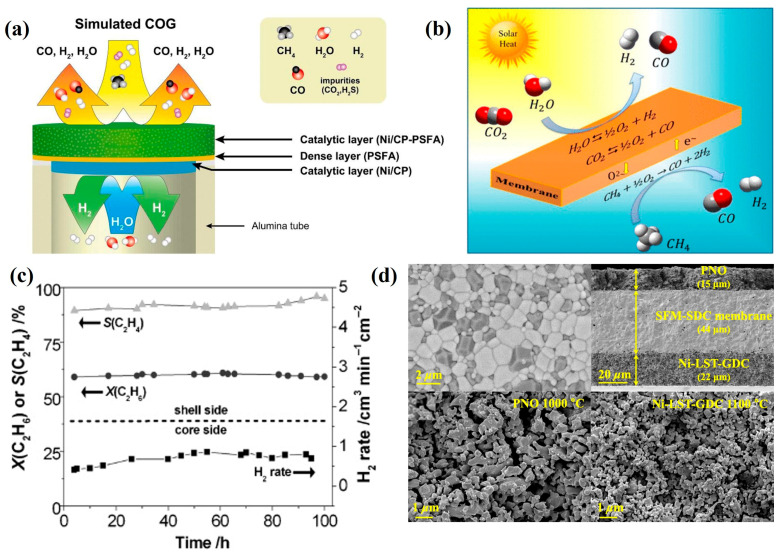
(**a**) The concept of hydrogen production using an asymmetric PSFA catalytic membrane reactor by coupling water splitting with COG oxidation [60]; (**b**) syngas production by splitting of H_2_O and CO_2_ [108]; (**c**) 100 h operation for H_2_ production on the core side and the simultaneous ethylene production on the shell side at 800 °C [111]; (**d**) SEM images of the coated membrane [83].

As displayed in Figure 9a, Ghanem et al. [60] engineered an asymmetric membrane reactor that integrates water decomposition with coke oven gas oxidation, achieving CO-free hydrogen production at a rate of 9.8 mL·min^−1^ cm^−2^ at 940 °C using a Ni/CP-catalyzed PSFA membrane. Remarkably, this configuration exhibited stable operation for over 250 h even in the presence of H_2_S and CO_2_ contaminants, showcasing the membrane’s exceptional tolerance to these common industrial gas impurities. Building upon this concept, Liang et al. [108] developed a CP-PSF membrane reactor system that simultaneously performs water splitting with CO_2_ co-feeding on one side and methane partial oxidation on the other. This dual-function reactor successfully produced syngas streams on both sides, with generation rates of 1.3 mL·min^−1^ cm^−2^ from the H_2_O/CO_2_ feed (at a ratio of 5) and 3.9 mL·min^−1^ cm^−2^ from the methane side when operated at 930 °C (as presented in Figure 9b). In a complementary approach, Zhang et al. [90] designed a YSZ-LSCF composite membrane that combines water splitting with CO_2_ capture functionality. The incorporation of the YSZ phase not only significantly improved the mechanical strength of the bilayer membrane but also ensured excellent long-term stability during approximately 290 h of continuous operation at 900 °C using syngas feed, with no observable performance degradation.

The production of C_2_ hydrocarbons via methane coupling reactions emerged in the 1980s with the development of novel materials such as SrCo_0.8_Fe_0.2_O_3−δ_ and SrCe_0.95_Yb_0.05_O_3−δ_ [109,110]. Jiang et al. [111] demonstrated a perovskite BCFZ oxygen transport membrane reactor capable of simultaneous hydrogen production via water splitting on one side and ethylene generation via oxidative dehydrogenation of ethane (C_2_H_6_ + O^2−^→C_2_H_4_ + H_2_O + 2e^−^) on the other. As illustrated in Figure 9c, this configuration achieved an ethane conversion rate of ~60% with 90% ethylene selectivity over 100 h of operation while maintaining a hydrogen production rate of 0.8 mL·min^−1^ cm^−2^ from water dissociation. A key advantage of this integrated system lies in the inherent spatial separation between hydrogen produced from water splitting and ethylene formed via dehydrogenation, preventing undesirable side reactions. Ammonia and liquid fuel synthesis represent two critical industrial processes that conventionally require substantial energy input for H_2_/N_2_ and H_2_/CO syngas production. Li et al. [112] developed an innovative BCF membrane reactor that combines these two syngas production steps into a single unit. By feeding H_2_O/air mixtures on one side and CH_4_ on the other, the reactor simultaneously generated ammonia synthesis gas (H_2_/N_2_) and liquid fuel synthesis gas (H_2_/CO). At 925 °C, optimized water-to-air ratios yielded production rates of 18.8 mL·min^−1^ cm^−2^ for ammonia synthesis gas and 45.6 mL·min^−1^ cm^−2^ for liquid fuel synthesis gas. This approach not only reduces energy consumption but also enhances process efficiency by integrating multiple catalytic conversions within a single membrane reactor system.

Current research on oxygen transport membranes for water splitting and methane partial oxidation primarily focuses on high-temperature operation (> 900 °C) to enhance surface exchange kinetics. Son et al. [83] reported an ultrathin SDC-SFM membrane reactor (44 μm thick) with a PNO coating (15 μm) and Ni-LST-GDC functional layer (22 μm), as illustrated in Figure 9d. This architecture demonstrated high ionic conductivity and chemical stability even at a reduced operating temperature of 800 °C, achieving stable hydrogen production (4.5 mL·min^−1^ cm^−2^) via water splitting and syngas generation (14 mL·min^−1^ cm^−2^) through methane conversion. A critical challenge for future development lies in designing membrane materials capable of maintaining structural and functional integrity under prolonged exposure to strongly reducing and corrosive environments.

## 5. Summary and Future Perspectives

Thermochemical water splitting utilizing oxygen transport membrane materials has garnered significant research attention due to its energy efficiency, environmental friendliness, and material recyclability. By leveraging the selective oxygen permeability of MIEC membranes, membrane reactor technology effectively overcomes thermodynamic equilibrium limitations inherent in conventional water splitting, substantially enhancing hydrogen production rates. This approach integrates reaction and separation processes into a single unit, offering superior efficiency, energy savings, and environmental benefits. When coupled with oxidative reactions such as methane partial oxidation, the system simultaneously produces high-value chemicals, further improving economic viability.

This review summarizes recent progress in oxygen transport membrane reactors for water splitting, focusing on membrane materials, reactor configurations, separation mechanisms, catalysts, and coupled reaction systems. For membrane materials, high oxygen flux and long-term stability remain paramount. Current research emphasizes the design and optimization of perovskite- and fluorite-structured single-phase/composite membranes, where elemental doping and structural engineering enhance oxygen permeability and chemical stability. Additionally, advances in membrane architectures (e.g., hollow fibers and porous-supported thin layers) and catalysts (e.g., Ni- and Ru-based systems) have significantly improved reaction kinetics.

Nevertheless, challenges persist, including insufficient long-term stability, high-temperature sealing difficulties, technological immaturity, and scalability limitations. Future research directions should prioritize the following areas:

(1) Development of novel membrane materials with balanced oxygen permeability, hydrogen production rates, and stability. High-entropy strategies show promise for enhancing thermodynamic stability through configurational entropy maximization. However, the increased complexity of multi-element doping necessitates extensive experimental screening to identify optimal OTM compositions.

(2) Optimization of membrane reactor geometries. To achieve low mass-transfer resistance, cost-effectiveness, and mechanical robustness, asymmetric membranes with ultrathin-dense layers on microchanneled supports represent a promising configuration. Future studies should systematically investigate structural optimization strategies.

(3) Integration of machine learning and multiscale modeling to accelerate material discovery and process optimization. Combining data-driven approaches with physics-based simulations can establish a closed-loop workflow from computational design to experimental validation, significantly shortening development cycles.

(4) Modularization and system integration for industrial deployment. Exploring low-temperature operation and contamination-resistant materials will enhance practical applicability. Through interdisciplinary collaboration and technological innovation, membrane reactors hold the potential for green hydrogen production.

Currently, MIEC membrane reactors for thermochemical water splitting remain at the laboratory stage. The development of advanced membrane materials exhibiting both high oxygen flux and operational durability represents the critical path toward industrial implementation. Addressing these challenges will require coordinated efforts across materials science, reaction engineering, and system design to unlock the full potential of this technology.
